# Likelihood of Leveraging Augmented Reality Technology to Promote HIV Prevention and Treatment Among Adolescent Girls and Young Women in Cameroon: Cross-Sectional Survey

**DOI:** 10.2196/69471

**Published:** 2025-05-12

**Authors:** Zhao Ni, Intan Maharani Sulistyawati Batubara, Jackson Jr Nforbewing Ndenkeh, Georges Bediang, Habakkuk Yumo, Xuehong Zhang, Sunyong Oh, Yuchen Zhao, LaRon E Nelson

**Affiliations:** 1 School of Nursing, Yale University, 400 West Campus Drive, Orange, CT, 06477, United States, 1 2037373039; 2 Center for Interdisciplinary Research on AIDS (CIRA), Yale University, New Haven, CT, United States; 3 Research for Development (R4D) International Foundation, Yaounde, Cameroon; 4 Faculty of Medicine and Biomedical Sciences, University of Yaounde, Yaounde, Cameroon; 5 Transatlantic Health Solutions LLC, Mesquite, TX, United States; 6 School of Literature, Media, and Communication, Georgia Institute of Technology, Atlanta, GA, United States

**Keywords:** adolescent, augmented reality, AR, mHealth, mobile apps, HIV/AIDS, PrEP, women, pre-exposure prophylaxis

## Abstract

**Introduction:**

Adolescent girls and young women in sub-Saharan Africa (SSA) represent 4 out of every 5 newly diagnosed HIV cases among adolescent girls and young women globally. Leveraging augmented reality (AR) technology for HIV prevention and treatment holds significant potential among young people. However, there is a knowledge gap regarding the acceptance of AR by adolescent girls and young women in SSA.

**Objective:**

This study aimed to assess the likelihood of adolescent girls and young women in Cameroon using AR for HIV testing, prevention, and treatment. The study findings will lay the groundwork for developing AR-based interventions to prevent and treat HIV in Cameroon and beyond.

**Methods:**

This was a cross-sectional survey conducted in Yaounde, Cameroon, in which 637 adolescent girls and young women were recruited using a combination of multistage cluster and snowball sampling techniques. We used an electronic survey to collect data on participants’ knowledge, prior use of AR technology, and likelihood of using AR technology for HIV prevention and treatment, and associated factors. Multivariate ordinal regressions were used to analyze the factors associated with the likelihood of adolescent girls and young women using AR to prevent HIV.

**Results:**

The study showed that 84% (536/637) of adolescent girls and young women had never heard of AR before this study, and only 8% (49/637) had prior experience using AR. Participants’ median age was 22 (IQR 21‐24) years, with the majority (362/637, 56.8%) aged between 21 and 25 years. Despite the low usage rate of AR among participants, there was a high likelihood of using AR to promote HIV prevention and treatment. Specifically, 72% (459/637) of participants reported that they were likely to use AR to visualize the HIV transmission process, while 73% (465/637) and 74% (471/637) reported the likelihood of using AR to learn about pre-exposure prophylaxis (PrEP) and how HIV medication lowers HIV viral load, respectively. More importantly, 54% (342/637) and 50% (319/637) of participants reported that they were extremely likely to use AR to learn the correct way of using condom and self-testing for HIV, respectively. The high likelihood of using AR to prevent and treat HIV was associated with a higher education level (*P*=.01), having ever tested for HIV (*P*=.03), and a history of previously using health apps or searching for health information on their phones (*P*<.001).

**Conclusions:**

The likelihood of using AR technology to promote HIV prevention and treatment is high among adolescent girls and young women in Cameroon. Future research should focus on exploring the preferred features of AR-based digital health interventions and consider methods of implementing them in the context of Cameroon or SSA.

## Introduction

In sub-Saharan Africa (SSA), the most vulnerable population for HIV is adolescent girls and young women [1]. Among adolescent girls and young women globally, 4 out of every 5 newly diagnosed HIV cases occur in SSA [2,3]. Cameroon, a lower-middle-income country with a population of 29 million, has the highest HIV prevalence rate in West Central Africa. Women, particularly those aged 15‐49 years, are disproportionately affected by the virus [4-7]. This health disparity among women is related to various factors, including limited access to health care resources, unbalanced power dynamics in sexual communication, reduced awareness of risk behaviors associated with HIV, and the absence of an enabling environment for women [8]. Consequently, the number of new infections among adolescent girls and young women in Cameroon is more than twice that of their male counterparts [9]. To eliminate the burden of HIV, the government of Cameroon has initiated national strategic plans aimed at reducing the HIV incidence by 65% by 2023 [7]. Although Cameroon is making progress in the fight against HIV, with a 50% decrease in HIV prevalence among people aged 15 to 64 years in the past 14 years, the 65% goal has not yet achieved [10]. This shortfall is associated with factors at individual, health system, and structural levels, including delays in diagnosis and linkage to HIV care, as well as insufficient engagement among persons who initiate care [11]. To end HIV in Cameroon, the national strategic plans of Cameroon emphasize the importance of providing adolescents and young people the right skills to protect themselves from HIV [7]. Under this backdrop, it is necessary to develop and test innovative, effective, and culturally appropriate approaches to enhance efforts in fighting against HIV in Cameroon, reduce health disparities, and promote HIV testing and pre-exposure prophylaxis (PrEP) use among adolescent girls and young women [12].

Innovative technologies, such as augmented reality (AR), have been integrated into mobile health (mHealth) interventions to promote user engagement, enhance interactive learning, and provide tailored health information [13]. AR is broadly defined as a technology, usable on devices such as smartphones, tablets, glasses, headsets, and so on, that overlays computer-generated information, such as avatars, images, and sounds, onto the users’ real environment in real time to enhance users’ interactive experience [13,14]. Additionally, a major technical limitation of conventional HIV prevention strategies is their lack of offering an interactive and immersive environment to enhance users’ experience [15-17]. To young people who are more open to technology, AR-based mHealth interventions could offer innovative solutions, through its immersive and interactive qualities, to enhance HIV health education, communication for behavioral change, and reduce stigma. For example, studies have found that AR applications have the potential to significantly increase user engagement, promote information retention, and enhance users’ satisfaction [18]. Furthermore, AR-based interventions have the capability to influence people’s perceptions of HIV prevention, potentially revolutionizing health education and behavior change initiatives, with youth as the primary target audience [19].

Despite their potential, most AR-based mHealth interventions have been conducted in high-income countries, leaving insufficient evidence of their applicability in low-income settings [20]. Some AR-based interventions, including “Floating heart” 3D imaging [21], mLearning [22], BodyExplorer [23], and FlexAR [24,25], have demonstrated preliminary but significant outcomes in medical education. However, their application to improve HIV prevention (including condom use and HIV PrEP uptake) is very sparse. Also, implementing AR interventions in resource-limited settings often faces technical challenges, including the need for smartphones with AR capabilities and reliable internet connections. A previous analysis has highlighted a high ownership of smartphones and access to the internet among adolescent girls and young women in Yaounde, Cameroon [17], indicating a favorable environment for implementing digital health interventions like AR-based mHealth programs. Understanding how adolescent girls and young women in Cameroon perceive and engage with AR technology is crucial for designing effective, culturally tailored health interventions. To date, the usability and effectiveness of AR technology in HIV prevention among adolescent girls and young women in Cameroon and the SSA region is unknown. Therefore, this study aimed to assess the likelihood of adolescent girls and young women using AR for the promotion of HIV testing, prevention, and treatment. This study also sought to identify factors associated with the likelihood of adolescent girls and young women adopting AR-based health interventions, informing the design of future AR-based mHealth interventions in Cameroon and the SSA region.

## Methods

### Study Sample and Data Collection

This was a cross-sectional study conducted from February to June 2023, in which 637 participants were recruited from Yaounde, Cameroon, where a variety of adolescent girls and young women were residing. To be eligible to participate in this study, individuals must be cisgender women aged 18 to 30 years, able to understand the study questions, and mentally healthy enough to voluntarily give informed consent.

The study procedures have been published elsewhere [17]. In brief, participants were recruited using a multistage cluster sampling technique based on Cameroonian health districts and health areas, combined with a snowball sampling technique wherein a consenting participant referred other potential participants. This recruitment method was chosen to ensure the inclusion of participants with diverse demographic backgrounds within Cameroon. Data were collected as pseudonymized data using a survey questionnaire implemented on Yale Qualtrics, a secure electronic survey platform. The Qualtrics questionnaire was administered with the assistance of 2 well-trained enumerators, who could hand their phones to participants to select their own responses, especially for sensitive questions. For reasons of data protection and confidentiality, participants’ identifier codes were only known to the 2 enumerators involved in fieldwork and JJNN, who possesses the signed inform consent forms.

### Ethical Considerations

This study received ethical approvals from the Yale University Institutional Review Board (ID: 2000033713) and the Cameroonian Centre Regional Ethics Committee for Human Health Research (initial ID: CE number 02058/CRERSHC/2022, and extension ID: CE number 02058/CRERSHC/2023). Additionally, this study has received administrative authorization from the Cameroonian Centre Regional Delegation for Public Health. Data were pseudonymized and all participants provided informed consent.

### Measures

#### Sociodemographic and Baseline Characteristics

Sociodemographic variables included age, monthly income, educational level, and geographical zone of origin. Other characteristics included sexual orientation, phone ownership and use, awareness of AR, and previous experience with AR. Specifically, phone ownership was measured by the question “Do you own or have access to the following devices on a daily basis (check all that apply: landline telephone, mobile phone without internet access, smartphone, tablet, laptop, and personal computer)?” Participants’ awareness of AR was measured by the question “Have you ever heard about Augmented Reality (Yes or No)?” For those who were aware of AR, their previous experience with AR was measured by the question “Have you ever used Augmented Reality (Yes or No)?” For participants who had used AR, a follow-up question was asked to study the features of AR that they used: “Why do you use Augmented Reality in your daily life? Select all that apply (Immersive, interactive, virtual world, embodiment, sensory feedback, other, please specify).”

#### Likelihood of Using AR

The primary outcome variable was the likelihood of using AR for health purposes, measured as a composite score of 7 subscales. The seven subscales measured the likelihood of leveraging AR to (1) visualize the HIV transmission process, (2) demonstrate the correct way of using a condom, (3) introduce daily PrEP, (4) depict the HIV testing process in a clinical or NGO setting, (5) show the HIV self-testing process, (6) explain the process of receiving HIV treatment, and (7) illustrate how HIV medication lowers the HIV viral load. Participants’ responses to each subscale were measured using a 5point Likert scale, ranging from 1 (extremely unlikely) to 5 (extremely likely). For example, the likelihood of leveraging AR to visualize the HIV transmission process was measured by the question: “If we build an Augmented Reality (AR) app for you to visualize the HIV transmission process, how likely would you be to use the AR*?*” and the likelihood of leveraging AR to demonstrate the correct way of using a condom was assessed by the question: “If we build an Augmented Reality (AR) app demonstrating the correct way of using condom, how likely would you be to use the AR?” A composite score was calculated for each participant as the mean score of the seven 5-point Likert subscales, rounded to the nearest whole number. Specifically, for each of the above subscales, we quantified their responses using a scale ranging from 1 to 5, followed by calculating the mean of these responses and rounded the results to the nearest whole number within the same Likert scale.

### Statistical Analyses

Statistical analyses were conducted using R software version 4.3.1 (R Core Team). Categorical variables were presented as absolute and relative frequencies, as well as cross tabulations of the likelihood categories to use AR. Proportions of the likelihood to use AR for HIV prevention were calculated, where the numerators were the number of participants who selected any of the ordinal response categories (ie, extremely unlikely, somewhat unlikely, neither likely nor unlikely, somewhat likely, and extremely likely), and the denominator was the total number of study participants. To determine factors associated with the likelihood of using AR for health purposes, ordinal logistic regression was applied using the “*polr*” function. Univariate analyses were initially conducted to identify covariates of interest (with *P*<.10), which were then put together in a model to adjust for each other’s effect in a multivariate analysis. The level of significance for the multivariate analysis was set at 5%. The exhaustive list of independent variables of the univariate analyses include: age group, region of origin, sexual orientation, education level, employment status, monthly revenue, marital status, housing condition, ownership of (or access to) smartphone or tablet with internet, history of health app use or phone for health information search, HIV testing situation, sexually transmitted infection diagnosis within past 6 months, vulnerable sexual behavior and PrEP awareness.

## Results

### Participant Characteristics

Participants’ median age was 22 (IQR 21‐24) years, with the majority (362/637, 56.8%) aged between 21 and 25 years. Most participants were heterosexual (599/637, 94%), unmarried (485/637, 76.1%), and were unaware of PrEP (519/637). We also found that 93.9% (598/637) of participants had access to a smartphone, 42.4% (270/637) had access to a laptop or personal computer, and 9.9% (63/637) owned a tablet or iPad [17]. Among the 637 participants, 84.1% (n=536) had never heard of AR, and only 7.7% (n=49) had used AR. Among those who had ever used AR, 51% (25/49) used virtual world, 20% (10/49) used embodiment features, 10% (5/49) used interactive elements, 10% (5/49) used sensory feedback, and 8% (4/49) used immersive features.

### Likelihood to use AR for Health Purposes and Associated Factors

Overall, the likelihood of using AR for health purposes was high, with 72.8% (464/637) of participants somewhat or extremely likely to use AR, compared to 10.5% (67/637) of participants somewhat or extremely unlikely to use AR. It should be noted that for each of the health purposes, at least 45% (287/637) of participants were extremely likely to use AR, with 53.7% (342/637) preferring to use AR for learning condom use and 50.1% (319/637) preferring to use AR for learning the process of HIV self-testing (Table 1).

**Table 1.** Participants’ likelihood to use augmented reality for health purposes (N=637).

Variable	Extremely unlikely, n (%)	Somewhat unlikely, n (%)	Neither likely nor unlikely, n (%)	Somewhat likely, n (%)	Extremely likely, n (%)
Likelihood to use AR^a^ if it was built to visualize the HIV transmission process	19 (2.9)	55 (8.6)	106 (16.6)	169 (26.5)	288 (45.2)
Likelihood to use AR if it was built to demonstrating the correct way of using condom	18 (2.8)	47 (7.4)	80 (12.6)	150 (23.5)	342 (53.7)
Likelihood to use AR if it was built to introduce daily PrEP^b^	19 (2.9)	59 (9.3)	96 (15.1)	155 (24.3)	308 (48.4)
Likelihood to use AR if it was built to demonstrate the HIV testing process in a clinical or NGO^c^ setting	17 (2.7)	60 (9.4)	99 (15.5)	150 (23.5)	311 (48.8)
Likelihood to use AR if it was built to demonstrate the HIV self-testing process	15 (2.4)	58 (9.1)	94 (14.8)	151 (23.7)	319 (50.1)
Likelihood to use AR if it was built to demonstrate the process of receiving HIV treatment	14 (2.2)	58 (9.1)	97 (15.2)	161 (25.3)	307 (48.2)
Likelihood to use AR if it was built to demonstrate how HIV medication lowers HIV viral load	14 (2.2)	57 (8.9)	95 (14.9)	162 (25.4)	309 (48.5)
Overall likelihood of using AR (a composite score of the above variables)	7 (1.1)	60 (9.4)	106 (16.6)	168 (26.4)	296 (46.5)

a AR: augmented reality.

b PrEP: pre-exposure prophylaxis.

c NGO: nongovernmental organization.

In unadjusted analyses of the ordinal logistic regression (Table 2), increasing likelihood to use AR for health purposes was associated with education level, sexual orientation, access to a smartphone or tablet, previous use of a health app or a phone for health-related information search, diagnosis of sexually transmitted infection within past 6 months, HIV testing, and awareness of PrEP.

**Table 2.** Factors associated with participants’ likelihood to use augmented reality for health purposes (N=637).

Variables		OR^a^ (95% CI)	*P*^b^ value	aOR^c^ (95% CI)	*P* value
Education level			<.001		.01
Uneducated or primary		1		1	
Secondary		3.3 (2.16‐5.01)		2.0 (1.27‐3.25)	
Higher		3.1 (1.89‐5.28)		1.6 (0.85‐2.88)	
Sexual orientation			.02		.047
Heterosexual or straight		1		1	
Bisexual or lesbian or other		0.5 (0.26‐0.91)		0.5 (0.28‐1.01)	
Employment status			.08		.24
Unemployed		1		1	
Employed		0.7 (0.5‐1.04)		0.8 (0.49‐1.2)	
Phone or Tablet with internet			.002		.48
No		1		1	
Yes		2.7 (1.46‐4.97)		1.3 (0.65‐2.46)	
History of health app use or phone health info search			<0.001		<0.001
No		1		1	
Yes		3.6 (2.28‐4.95)		2.2 (1.41‐3.42)	
HIV testing			<0.001		.03
Never		1		1	
>12 months		1.2 (0.85‐1.71)		1.2 (0.8‐1.7)	
≤12 months		2.02 (1.4‐2.89)		1.7 (1.14‐2.62)	
STI^d^ in the past 6 months			.007		.21
No		1		1	
Yes		1.7 (1.15‐2.42)		1.3 (0.86‐1.98)	
Aware of PrEP			.002		.18
No		1		1	
Yes		1.9 (1.26‐2.77)		1.3 (0.88‐2.06)	

a OR: odds ratio

b Only variables with *P*<0.1 in univariate analaysis are shown in this table.

c aOR: adjusted odds ratio.

d STI: sexually transmitted infection.

Upon adjusting for all key covariates in a multivariate model, an increasing likelihood to use AR for health purposes was independently associated with a higher level of education (*P*=.01), having used a health-related app before or using a phone to search for health-related information (aOR 2.2, 95% CI 1.41‐3.42; *P*<.001), and having tested for HIV, notably less than 12 months ago (aOR 1.7, 95% CI 1.41‐2.62; *P*=.03), compared to those who have never undertaken an HIV test. Additionally, a decreasing likelihood to use AR for health purposes was independently associated with participants who identified as sexual minorities (aOR 0.5, 95% CI 0.28‐1.01; *P*=.04), compared to heterosexual participants.

## Discussion

### Principal Findings

The integration of AR into HIV prevention strategies holds promise for enhancing education, improving accessibility, and ultimately reducing the spread of HIV. However, there is a knowledge gap regarding how AR technology, which garners increasing interest worldwide, can be best used for HIV prevention in resource-limited settings. This study seeks to address the literature gap on the feasibility of using AR technology for HIV prevention among adolescent girls and young women in the Cameroonian context. The findings of this study have provided valuable information on the likelihood of Cameroonian adolescent girls and young women to use AR to learn about HIV testing, prevention, and treatment. Overall, there was a low prior knowledge of AR among adolescent girls and young women, accompanied by an even lower prior use of AR technology. Despite this, the likelihood of adolescent girls and young women using AR was high, with at least 45% indicating they were extremely likely to use AR interventions for HIV testing, prevention, and treatment, such as acquiring knowledge about HIV transmission, HIV testing, self-testing processes, correct condom use, HIV treatment, daily PrEP, and information on how HIV medication reduces viral load. This finding is particularly significant given that a study conducted in the Northwest region of Cameroon showed a high prevalence of inconsistent condom use among young women with HIV [26]. To end HIV in Cameroon and SSA, more comprehensive educational programs on sexual and reproductive health for adolescent girls and young women, including distribution and empowerment of female condom use, are necessary.

This study also indicated that leveraging AR is worth exploring in Cameroon. Globally, the awareness and use of AR for adolescent girls and young women in resource-limited settings is low compared to their counterparts in high-income settings [27]. This is mainly due to differences in access to AR technology, internet connectivity, and the integration of AR in education and entertainment [27]. Despite this, there have been a few commendable initiatives on the use of AR in SSA, notably in medical training, where it has shown to enhance the learning process and performance through more interactive and engaging experiences [28,29]. These initiatives, along with our study finding on the high likelihood of using AR among adolescent girls and young women, indicate that leveraging AR for HIV prevention in SSA is possible and should be given serious consideration.

This consideration is further justified by the interactive nature of AR technology. Compared with many early mHealth interventions, mobile apps embedded with AR technology can provide an immersive, interactive learning environment for people to receive real-time information, motivation, and behavioral skills. Considering the widespread adoption of smartphones among young people in resource-limited settings and the permeation of digital technologies in developing countries, AR-based mobile apps have significant potential for facilitating behavior change communication in HIV prevention efforts. For example, in a context where the ability of adolescent girls and young women to negotiate condom use during sexual acts is greatly influenced by a lack of comprehensive knowledge on HIV and cultural and social norms [30-32], AR technology, through interactive and educative role-play scenarios, may enhance their ability to effectively negotiate and correctly use condoms. Despite the lack of previous research on AR for HIV prevention among adolescent girls and young women, we believe that the expanded use and integration of technology-delivered interventions, such as mobile apps, websites, live chats, chatbots, vending machines, virtual reality and AR, when guided by theoretical frameworks, can assist adolescent girls and young women in engaging in self-care for HIV testing and prevention [33,34]. For instance, in 2018, the Joint United Nations Programme on HIV/AIDS (UNAIDS) teamed up with Google and Makhulu Media to release a series of virtual reality educational films on HIV testing. These films were distributed in clinics, schools, and communities across South Africa to encourage people to test for HIV, potentially reshaping attitudes and reducing HIV-related stigma [35].

Our study has identified several factors associated with the likelihood of adolescent girls and young women to use AR technology for health purposes. We observed that adolescent girls and young women with at least secondary education level were more likely to use AR for HIV prevention than those with less education. This might be because individuals with higher levels of education tend to have better digital literacy skills as education provides opportunities for engagement with digital technologies and developing necessary digital competencies [36,37]. We also observed that those who had previous exposure to mHealth apps and/or sought health-related information on mobile devices were more likely to accept AR use compared to those who did not use health apps or use their phones for health information inquiries. Given that mobile devices are integrated into everyday life, offering numerous opportunities to enrich the learning experience, prior exposure to smartphones in learning activities might have made them familiar and comfortable with technology-based health interventions [38].

In this study, we also found that adolescent girls and young women who had undergone HIV testing within the past 12 months had a higher likelihood of using AR for HIV prevention compared to those who had never been tested. This increased likelihood might be attributed to the counseling and education services related to HIV testing, which have raised awareness of HIV vulnerability and the preventive behavioral changes that should be adopted, thus increasing their openness to the potential role of AR technology in HIV prevention. Additionally, adolescent girls and young women who identified as sexual minorities (bisexual, lesbian, or other), compared to heterosexual, exhibited lower likelihoods to use AR for HIV-related services. This may be due to a lack of tailored HIV prevention that specifically address the needs and concerns of sexual minorities given the legal and social environment around same-sex relationships in Cameroon. It could also be due to stigma and discrimination based on sexual orientation, which may create barriers to accessing health care services, thus influencing the health care utilization and behavior of young female sexual minorities [39]. Furthermore, cultural and societal norms within certain communities in Cameroon may marginalize such populations [40].

### Limitations

Although our study has contributed valuable knowledge to the field of leveraging AR in Cameroon to prevent HIV among adolescent girls and young women, its findings should be applied with consideration of the following limitations. First, although we used multistage cluster sampling that randomly selected 4 health districts followed by the selection of at least 2 health areas from each health district, which increased the representation of adolescent girls and young women from the various regions of Cameroon based in Yaounde, our study sample was ultimately recruited using the snowball technique, relying on referrals from participants’ personal networks. This limits the generalizability of our study findings to all regions of Cameroon notably semiurban and rural areas which potentially lack the infrastructure to effectively run digital interventions. Second, while investigating their likelihood of using AR technology, we provided participants with an AR definition but did not provide an actual AR device or intervention; therefore, participants’ understanding of AR technology might be limited to only a conceptional level rather than with deeper user experience. This might have brought some biases within participant feedback and responses. Nonetheless, building on the findings of this study, our team aims to develop an AR-based mHealth intervention in the future to promote HIV prevention among adolescent girls and young women within the metropolitan cities of Cameroon.

### Conclusions

Our study reveals that the likelihood of leveraging AR for HIV prevention among adolescent girls and young women in Cameroon is high, particularly among those with higher educational levels and those who have previously used mobile apps for health purposes. The findings lay the foundation for the necessity of developing and testing AR-based interventions to prevent HIV in Cameroon. These interventions should be based on behavioral theories, Cameroonian culture, and the preferences of adolescent girls and young women in Cameroon.

## Abbreviations

AR: augmented reality
mHealth: mobile health
PrEP: pre-exposure prophylaxis
SSA: sub-Saharan Africa
UNAIDS: Joint United Nations Programme on HIV/AIDS
